# At the dawn: cell-free DNA fragmentomics and gene regulation

**DOI:** 10.1038/s41416-021-01635-z

**Published:** 2021-11-23

**Authors:** Yaping Liu

**Affiliations:** 1 Division of Human Genetics, Cincinnati Children’s Hospital Medical Center, Cincinnati, OH 45229 USA; 2 Division of Biomedical Informatics, Cincinnati Children’s Hospital Medical Center, Cincinnati, OH 45229 USA; 3 Department of Pediatrics, University of Cincinnati College of Medicine, Cincinnati, OH 45229 USA; 4 Department of Electrical Engineering and Computing Sciences, University of Cincinnati College of Engineering and Applied Science, Cincinnati, OH 45229 USA

**Keywords:** Cancer genomics, Computational biology and bioinformatics, Epigenomics, Gene regulation

## Abstract

Epigenetic mechanisms play instrumental roles in gene regulation during embryonic development and disease progression. However, it is challenging to non-invasively monitor the dynamics of epigenomes and related gene regulation at inaccessible human tissues, such as tumours, fetuses and transplanted organs. Circulating cell-free DNA (cfDNA) in peripheral blood provides a promising opportunity to non-invasively monitor the genomes from these inaccessible tissues. The fragmentation patterns of plasma cfDNA are unevenly distributed in the genome and reflect the in vivo gene-regulation status across multiple molecular layers, such as nucleosome positioning and gene expression. In this review, we revisited the computational and experimental approaches that have been recently developed to measure the cfDNA fragmentomics across different resolutions comprehensively. Moreover, cfDNA in peripheral blood is released following cell death, after apoptosis or necrosis, mainly from haematopoietic cells in healthy people and diseased tissues in patients. Several cfDNA-fragmentomics approaches showed the potential to identify the tissues-of-origin in cfDNA from cancer patients and healthy individuals. Overall, these studies paved the road for cfDNA fragmentomics to non-invasively monitor the in vivo gene-regulatory dynamics in both peripheral immune cells and diseased tissues.

## Main

Multidimensional epigenetic layers play instrumental roles in mammalian gene regulation [1–3]. During cancer initiation and progression, embryonic development and organ transplantation, epigenomes vary across different time points and impact gene expression and biological function [4–9]. In animal models, the variations of the epigenome and transcriptome across different time points can be characterised through high-throughput sequencing technologies, such as ATAC-seq and RNA-seq, by sacrificing the animals with the same genetic background [10–12]. In cultured human cells still alive, the epigenome dynamics can be visualised by fluorescence live imaging [13–15]. However, in human subjects still alive, especially for the inaccessible human tissues, such as solid tumours, fetuses during pregnancy and transplanted organs, how to non-invasively monitor the dynamics of their epigenomes and transcriptomes is still largely unexplored.

Circulating cell-free DNA (cfDNA) in the peripheral blood offers a promising and non-invasive approach to monitoring the genome dynamics from the inaccessible human tissues [16–18]. CfDNA molecules from the tumours, foetus and transplanted organs can be separated out based on the allelic status of mutations or single-nucleotide polymorphisms (SNPs) within fragments measured through deep whole-genome sequencing (WGS). Thus, part of or the whole genome in these inaccessible tissues can be reconstructed across different time points [19–21]. However, most of the current cfDNA WGS studies only focus on the genetic aberrations in the cfDNA, such as SNPs or copy number variations (CNVs) [22–24], but not epigenomes. Since the cfDNA fragments are outside the cells and highly fragmented, it is challenging to directly characterise the epigenomes, except for covalent DNA modifications and very few other cases [25–31]. Interestingly, the fragmentation patterns of cfDNA are not uniform across the genome [32, 33]. Several studies suggested a tight correlation between cfDNA-fragmentation patterns and in vivo gene-regulatory maps within the cells, such as nucleosome positioning and gene expression [32–34]. These correlations raise the possibility of inferring back the epigenome and transcriptome within the cells from the cfDNA-fragmentation patterns. In this review, we will first go through the major milestones of cfDNA-fragmentation studies in the pre-omics era, which inspired many studies in the later-omics era. Further, we will summarise the state-of-art high-throughput methods, mostly computational, on the measurement of cfDNA fragmentation (Table 1). Finally, we will go through the approaches for the tissue-of-origin analysis using cfDNA fragmentation.

**Table 1:** Summary of the cfDNA fragmentomics across different resolutions.

Methods	sequencing-based	Resolution	Reported/potential applications	Performance reported for cancer diagnosis	Tissue-of-origin inference	Potential limitations
Large-scale fragmentation patterns at megabase level (DELFI) [64]	Yes	5 Mb	Diagnosis of multiple early-stage cancers	57% to >99% sens@98% spec, AUC 0.94	Distinguish different cancer types (supervised way)	Performance is only evaluated by cross-validation. lack of a large independent prospective cohort for the validation. control is not age-matched. No application to the non-oncology field. low resolution limited the follow-up study to locate the potential therapeutic targets.
Large-scale co-fragmentation patterns (FREE-C) [65]	Yes	250 kb–1Mb	Cancer diagnosis (potential)	—	Absolute contribution value from different cell types	Lack of benchmark on the cancer diagnosis. low resolution limited the follow-up study to locate the potential therapetic targets. the reference panel for the tissues-of-origin estimation is arbitrary.
Fragment coverage near TSS [34]	Yes	±2 kb	Cancer diagnosis (potential)	—	—	Lack of benchmark on the cancer diagnosis. the gene expression prediction is only on a limited number of genes (housekeeping and always silenced) with binary prediction.
cfDNA-accessibility score near the transcription factor-binding sites (TFBS) [66]	Yes	±1 kb	Diagnosis of early-stage colon cancer	71% sens@72% spec for stage I, 74% sens@77% spec for stage II	Distinguish different cancer types (supervised way)	Performance is only evaluated by cross-validation. lack of a large independent prospective cohort for the validation. control is not age-matched. no application to the non-oncology field.
Orientation-aware cfDNA fragmentation (OCF) [67]	Yes	±1 kb	Diagnosis of early-stage HCC, organ transplantation, pregnancy	67.6% sens@ 93.8% spec	Estimate relative contributions from several cell types	Depending on the known open-chromatin regions. performance is only evaluated by cross-validation. lack of a large independent prospective cohort for the validation. control is not age-matched.
Windowed protection score (WPS) [33]	Yes	±120 bp	Cancer diagnosis (potential)	—	Estimation of several relevant cell types	Lack of benchmark on the cancer diagnosis. accurate nucleosome inference largely depends on the deep sequencing. many parameters in tissues-of-origin estimation are arbitrary
cfDNA-fragmentation hotspots [68]	Yes	200 bp	Diagnosis of multiple early-stage cancers	42% to 93% sens@100% spec	Distinguish different cancer types (supervised way)	Performance is only evaluated by cross-validation. lack of a large independent prospective cohort for the validation. control is not age-matched. no application to the non-oncology field.
Inference of DNA methylation from cfDNA fragmentation patterns [69]	Yes	Single base pair (high coverage) or 1 kb (low coverage)	Cancer diagnosis (potential)	—	Absolute contribution value from different cell types	Inference accuracy and resolution is the concern. lack of clinical applications in the study.
Preferred-ended position of cfDNA [70, 71]	Yes	Single base pair	Diagnosis of early-stage HCC, organ transplantation, pregnancy	AUC 0.88	Estimation of most relevant cell types	Accurate estimation of preferred-end sites requires deep sequencing. performance is only evaluated by cross-validation. lack of a large independent prospective cohort for the validation. control is not age-matched.
End-motif frequency and motif-diversity score (MDS) [72]	Yes	Global summary statistics	Diagnosis of multiple cancers, organ transplantation, pregnancy	AUC 0.89 (end motif), AUC 0.85 (MDS)	Estimation of most relevant cell types	MDS and end motif are summary statistics, their relationship with gene regulation at different location is not clear. performance is only evaluated by cross-validation. Lack of a large independent prospective cohort for the validation. Control is not age-matched.
Jagged end [73, 74]	Yes	Fragment level	Diagnosis of HCC, pregnancy	AUC 0.87 (JI-U in fragments 130–160 bp), AUC 0.54 (JI-U for all fragments)	Estimation of most relevant cell types	Performance is only evaluated by cross-validation. lack of a large independent prospective cohort for the validation. control is not age-matched.
Fragmentation patterns at eccDNA [76–78]	Yes	—	Cancer diagnosis (potential), pregnancy	—	—	Lack of benchmark on the cancer diagnosis and other studies.
LIQUORICE [131]	Yes	Integration of multiple resolutions	Diagnosis of Ewing sarcoma and other paediatric sarcomas	AUC up to 0.97	—	Fragmentation pattern in fine-scale depending on the known open-chromatin regions. cases are paediatric cancer, while healthy controls are all adults from other cohorts. lack of a large independent prospective cohort for the validation.
Enrich tumour signals by fragment size [60, 85]	Yes	Fragment level	Pan-cancer diagnosis	AUC 0.99 (late stage)	—	Control is not age-matched. sample size is small in each cancer category. cancer samples are late stage.
Filter CHIP-associated variants by fragment size [89, 90]	Yes	Single variant	Pan-cancer diagnosis (INVAR), early-stage lung cancer	AUC 0.98 (late stage, INVAR), 0.80 (early-stage, INVAR); 41% to 67%@98% specificity (Lung-CLiP, stage 1–3, risk matched control, independent prospective validation)	—	Classification is mostly based on genetic variants. control is not age-matched (INVAR). sample size is small in each cancer category (INVAR). Specialised sequencing technology (Lung-CLiP + CAPP-seq)
qPCR based	No	—	Cancers diagnosis, organ transplantation, pregnancy	—	—	Not genome-wide
Sophisticated capillary electrophoresis	No	—	Cancers diagnosis	—	—	Not genome-wide
Microscopy based	No	—	Cancers diagnosis	—	—	Not genome-wide

Non-NGS based methods are not discussed in detail.

## The pre-omics era of cfDNA-fragmentation studies

Early in 1948, the presence of cell-free nucleic acids in the circulation was first described by Mandel and Metais in human blood from healthy individuals and patients with different diseases [35]. In the beginning, circulating cell-free DNA (cfDNA) was demonstrated to be predominantly double-stranded [36]. Later in 1973, single-stranded cfDNA fragments were also characterised in the serum from systemic lupus erythematosus (SLE) patients [37]. The variations of strandness and sizes of cfDNA fragments altered the clearance kinetics and mechanism of DNA in the circulation [38]. Stroun et al., in 1987, showed that cfDNA from cancer patients was double-stranded primarily with the size of 0.5–21 kb and revealed that cfDNA from cancer patients is smaller than genomic DNA with fragmented status [39]. Later, several studies suggested that cfDNA is mainly released from apoptotic or necrotic cells [40, 41], which is further proved by multiple subsequent studies [42, 43]. In 2002, Lui et al. first demonstrated that the cfDNA from healthy individuals mostly comes from the haemopoietic cells by estimating the percentage of cfDNA from the Y chromosome in the plasma from sex-mismatched bone marrow transplantation patients [44]. In 2003, Deligezer et al. suggested a significant correlation between the fragmentation status in cfDNA and DNA methylation, as well as the nucleosomes, at the 1st exon of the p16 gene in lymphoma patients [45]. Further, the study of variations in cfDNA-fragment sizes was expanded to other fields, such as prenatal test [46].

Before the development of next-generation sequencing (NGS) technology, quantitative PCR (qPCR) was applied to study the fragmentation in cfDNA. By using qPCR, Diehl et al. discovered that mutant sequences are enriched in small DNA fragments (<180 bp) but not in long fragments [40, 47, 48]. Further, by optimised qPCR and xenografted model, Mouliere et al. first indicated that cancer-derived cfDNA showed higher fragmentation as compared with healthy controls highlighting higher fragmentation as a hallmark of cancer cfDNA (circulating tumour DNA, ctDNA) [49]. Sanchez et al. systematically compared the distribution of cfDNA-fragment sizes with cfDNA WGS in both double-stranded and single-stranded cfDNA by qPCR and discussed the association of mononucleosomes and chromatosomes with cfDNA [50].

Moreover, Meddeb et al. quantified the influence of several pre-analytical and demographic parameters on the overall variations of fragmentation in nuclear and mitochondrial circulating cfDNA [51]. Recently, droplet digital PCR (ddPCR) was combined with bisulfite treated by Shemer et al. to accurately quantify the tissues-of-origin in cfDNA [52]. Other approaches, such as sophisticated capillary electrophoresis, electron microscopy, Raman microscope, 3D laser-scanning confocal microscope and atomic force microscopy, were also utilised to study the cfDNA fragments besides NGS [53–55]. However, due to the limitation of technologies, cfDNA-fragmentation studies at the pre-omic era were mostly limited to the summary statistics or limited loci in the genome. The correlation between genome-wide fragmentation patterns in cfDNA and gene regulation within the cells is still not explored.

## The cfDNA-fragmentomics era and gene regulation

Thanks to the advances in NGS, it is possible to measure the fragmentation patterns in millions of cfDNA molecules across different genomic locations in a high-precision and high-throughput way. Due to the overall short-fragment sizes in cfDNA, WGS library construction on cfDNA does not require the mechanic sonication step, which was widely used for the traditional WGS library construction on genomic DNA and will significantly affect the measurement of fragmentation patterns in cfDNA. Moreover, instead of a single summary-statistic score or characteristics from a limited number of loci, researchers can accurately estimate the fragment size, ends and strandness at every single cfDNA molecule, as well as the fragment coverage at each base of the reference genome [16, 56–61] [16, 56–59]. In 2008, Fan et al. identified the well-positioned nucleosomal patterns around TSS in plasma cfDNA but not in genomic DNA control by utilising the fragment coverages estimated from NGS [62]. In 2010, Fan et al. further demonstrated that the fragment size originated from the foetal side is smaller than those originated from the maternal side through paired-end NGS [63]. The concept of ‘cfDNA fragmentomics’ was first introduced by Ivanov et al. in 2015 [32]. Since then, in addition to the study of the fragment coverages and sizes, several innovative computational and experimental approaches have been developed to comprehensively measure the cfDNA-fragmentation patterns in plasma across different resolutions, including large-scale fragmentation patterns at megabase level (DELFI) [64], large-scale co-fragmentation patterns (FREE-C) [65], fragment coverage near transcription-start sites (TSS) [34], cfDNA-accessibility score near the transcription factor-binding sites (TFBS) [66], orientation-aware cfDNA fragmentation (OCF) [67], windowed protection score (WPS) [33], cfDNA-fragmentation hotspots [68], inference of DNA methylation from cfDNA-fragmentation patterns [69], the preferred-ended position of cfDNA [70, 71], the end-motif frequency and motif-diversity score (MDS) [72], jagged end [73, 74] and patterns outside the chromosomes [75–78]. Here, we will go through these state-of-art approaches applied at cfDNA fragmentation from a large-scale genomic bin to a single fragment.

### Large-scale fragmentation patterns (DELFI) and chromatin organisations [64]

“DNA evaluation of fragments for early interception” (DELFI) was developed to detect genome-wide fragmentation abnormality of cfDNA by ~1X low-coverage whole-genome sequencing (WGS). DELFI evaluated the fragment coverage, size and other summary statistics within 100-kb non-overlapping bins and aggregated them into the 5-megabase (Mb) non-overlapping window, which will bring more than 20,000 reads per window at 1–2X genome coverage. They estimated the ratio between short cfDNA fragments (100–150 bp) and long cfDNA fragments (151–220 bp) within each window and found the increased aberrations at cancer patients, not at healthy individuals. They further utilised these summary statistics for the diagnosis of early-stage cancer. The fragment coverage among these statistics in the window was the most critical classification feature in their stochastic gradient-boosting model. Regarding the correlation with gene regulation, they utilised the window-protection score (WPS) [33] approach to infer the nucleosomal positioning in both cfDNA- and DNase-digested genomic DNA (gDNA) from lymphocytes. The median distance of the nucleosome from cfDNA showed a high correlation with that from DNase-digested gDNA and intermediated correlation with the chromatin compartment characterised by Hi-C from lymphocytes. These results indicated the important role of both nucleosome occupancy and high-ordered chromatin organisations for the cfDNA fragmentomics at cancers.

### Large-scale co-fragmentation patterns (FREE-C) and 3D genome [65]

‘FRagmentation Evaluation of Epigenetics from CfDNA sequencing’ (FREE-C) was developed to evaluate the co-fragmentation patterns between pairs of large-scale genomic bins in the low-coverage WGS. They hypothesised that the cfDNA molecules released from two genomic regions would show similar fragmentation patterns at large scale if the two regions are spatially close to each other inside the cells, further informing the 3D genome status. There are two different approaches in FREE-C: multi-sample FREE-C and single-sample FREE-C. (1). In multi-sample FREE-C, they divided the chromosomes into 500-kilobase (kb) non-overlapping bins and calculated a ‘normalised fragmentation score’ based on fragment size at each bin for each individual. They then calculated the Pearson correlation coefficient of the fragmentation scores between each pair of bins in the same chromosome across all individuals. (2). Single-sample FREE-C calculated the fragment-size distributions of cfDNA in each genomic bin at a single cfDNA WGS sample. Further, FREE-C characterised the distance of the fragment-size distribution between each pair of genomic bins in the same chromosome using the Kolmogorov–Smirnov test. After obtaining the two-dimensional distance-correlation matrix between bins in each chromosome by multi-sample FREE-C and single-sample FREE-C, they found a high similarity with the correlation matrix characterised from Hi-C data in white blood cells (WBCs). The first eigenvector of the matrix from cfDNA in both approaches showed high similarity with the A/B compartments characterised from Hi-C data in WBCs. The fragment sizes of cfDNA are affected by multiple epigenetic backgrounds, such as DNA methylation and histone modifications [32, 79]. Thus, they performed the multivariate analysis and confirmed that the 3D genome signal is the major contributor to the co-fragmentation patterns in large-scale rather than sequence-composition bias or other large-scale epigenetic signals, such as CpG methylation and H3K4me1. This study has not been peer-reviewed and is supported by commercial entities.

### Fragment coverage near TSS and gene expression [34]

In 2016, Ulz et al. developed a computational approach to summarise the fragment coverages at two focal regions near TSS: 2000-bp region centred on the TSS (2K-TSS coverage) and −150 bp to +50 bp with respect to the TSS (NDR coverage). They normalised the fragment coverage for both regions with the relative copy number to exclude the potential effect of copy number alterations (CNAs) often observed in cancer. Further, Ulz et al. utilised these two features by a support-vector machine model for the binary classification of the housekeeping genes and constitutively not-expressed genes and achieved high accuracy in the cross-validation. Moreover, the model was applied in the cancer patients to predict the gene’s binary expression status or even several isoforms inside the tumour, suggesting a promising opportunity to non-invasively investigate the in vivo gene expression status at inaccessible tissues. The earlier study by Ivanov et al. [32] also revealed the associations between gene expression and nucleosome-fragmentation patterns near promoters but with whole-exome sequencing, which limited its capability to predict the gene expression status with high accuracy.

### The cfDNA-accessibility score and the binding affinity of the transcription factors [66]

In 2019, Ulz et al. developed an accessibility score to estimate the overall binding affinity of the transcription factor (TF) by cfDNA WGS. They summarised the cfDNA-fragment coverages near each transcription factor-binding site (TFBS). The gDNA-accessibility pattern near TFBS was different in proximal and distal regions to the TSS. They found the low-frequency pattern of cfDNA-fragment coverage at proximal TFBS and the high-frequency pattern of cfDNA-fragment coverage at distal TFBS. They utilised Savitzky–Golay filters to suppress effects on the coverage not contributed by nucleosome positioning and remove the local biases. Further, the high-frequency signals were ranked and finally utilised as the accessibility score. Ulz’s cfDNA-accessibility score around the cell-type-specific TFs showed similar overall trends as that at gDNA accessibility measured by ATAC-seq between cancer and healthy. Thus, they concluded that the cfDNA-accessibility score indicated the global in vivo binding affinity of TFBS in each cfDNA sample. Further, Ulz et al. demonstrated the ability of the cfDNA- accessibility score to distinguish the subtypes of late-stage prostate cancer by aggregated low-coverage cfDNA WGS. Finally, the accessibility score across 504 TFs showed good performance in identifying the early-stage colorectal-cancer samples even with the low tumour fraction.

### OCF and open-chromatin regions [67]

Sun et al. developed a computational approach to quantify the cfDNA-fragmentation patterns near the tissue-specific open-chromatin regions identified in previous epigenomic profiling by DNase-seq. First, they identified the tissue-specific open-chromatin regions from different publicly available DNA- accessibility data. Second, around the centre of these open-chromatin regions (±1000 bp), they calculated the coverage of upstream (U) fragment ends (the end mapped to the reference genome with a smaller coordinate), and the coverage of downstream (D) fragment ends (the end mapped to the reference genome with larger coordinate), respectively. Since the U and D ends showed peaks at −60 and +60 bp from the centre of the open-chromatin regions, respectively, they calculated the OCF value by quantifying the differences of coverages of the U and D ends in 20-bp windows around these two peaks for each tissue-specific open-chromatin regions. Naturally, the OCF value can be utilised to rank the relative contributions of different cell types to the circulating cfDNA and further application for the diagnosis of different pathological and physiological conditions.

### WPS, single-stranded cfDNA, nucleosomes and other epigenetic backgrounds [33]

In the traditional WGS library-preparation approach, damaged and short double-stranded DNA molecules were poorly recovered. Thus, Snyder et al. developed a single-stranded cfDNA library- preparation method, which was adapted from studies of damaged ancient DNA [80]. Several follow-up studies on single-stranded cfDNA further revealed that most cfDNA is highly nicked, which might be subjected to continuous nuclease activity in the bloodstream [50, 67, 81]. Snyder and his colleagues also developed a computational approach, named WPS, to reconstruct the footprint of nucleosome positioning and transcription factor binding through deep WGS. Basically, WPS was calculated by the number of DNA fragments completely spanning a genomic window minus the number of fragments with an endpoint within that same window. They applied a peak-calling algorithm on the genome-wide WPS signals and identified many nucleosome-occupied regions, which showed similar genomic distributions as the nucleosome maps previously identified within the blood cells. The long-fraction WPS (L-WPS) was calculated by long fragments (120–180 bp) with a larger window (120 bp) and short-fraction WPS (S-WPS) was estimated by short fragments (35–80 bp) with a smaller window (16 bp). While L-WPS captured the signal from nucleosome occupancy, S-WPS represented the potential of TF binding, such as CTCF. Further, the nucleosomal spacing identified by WPS showed correlations with the A/B compartments identified by Hi-C, open-chromatin regions and gene expression specifically from haematopoietic cells, which enable its potential to identify the tissues-of-origin from cfDNA.

### CfDNA-fragmentation hotspots, open-chromatin regions and transposons [68]

Cell-free dna fragmentation (CRAG) was developed to de novo characterise the genome-wide cfDNA- fragmentation hotspots in cfDNA WGS. Many studies suggested that local nucleosome structure reduces the fragmentation process, indicating the potential enrichment of cfDNA-fragmentation hotspots (lower coverage and smaller size) at the open-chromatin regions. Basically, they utilised a 200-bp sliding window to scan the genome. The fragment coverage was weighted by the ratio of average fragment sizes in the window versus that in the whole chromosome, named integrated fragmentation score (IFS). They further applied a negative binomial model to test if the windows showed significantly lower IFS than the local (5-kb and 10-kb centre to the window) and global background (whole chromosome). In cfDNA from healthy, cfDNA-fragmentation hotspots were enriched in gene-regulatory elements, including promoters, haematopoietic-specific enhancers and 3′ end of transposons. In cfDNA from early-stage cancers, IFS showed aberrant patterns at hotspots near microsatellites, CTCF and promoters of genes enriched in immune processes from peripheral immune cells. They also applied the IFS signals from hotspots for the diagnosis of multiple early-stage cancers with high accuracy. This study has not been peer-reviewed.

### Inference of DNA methylation from cfDNA-fragmentation patterns [69]

The cfDNA-fragment sizes are significantly different at methylated and unmethylated fragments [45, 79]. Liu et al. developed a machine-learning approach to infer the base-pair resolution DNA methylation level by the fragmentation patterns from cfDNA WGS [69]. Fragment size, coverage and the distance of each CpG to the fragment end in each fragment were utilised as features for a non-homogeneous hidden Markov model. The emission probability was estimated by adding a Bayesian factor of cfDNA methylation level from healthy peripheral blood mononuclear cells to the multivariate Gaussian mixture model. In cfDNA WGBS, with the ground truth of the binary CpG methylation status in each CpG at each fragment, they achieved about 0.92 area under the curve in the balance-sampled CpG sites at CpG-rich regions (>=10 CpGs in each fragment). They also achieved a high correlation in a 1-kb window between the prediction from cfDNA WGS by the matched cfDNA WGBS from the same tube of blood in both healthy and multiple cancer patients with deep and shallow sequencing. Using hundreds of WGBS datasets from different tumour and normal cells as the reference map, they deconvoluted cfDNA’s tissue-of-origin status by inferred DNA methylation level at ultra-low-pass WGS from thousands of breast or prostate cancer samples and healthy individuals. The tissue-of-origin status in cancer patients showed a high concordance with confirmed metastasis tissues from physicians and correlation with some clinical metadata. This study has not been peer-reviewed.

### The preferred-ended position of cfDNA [70, 71]

Taking advantage of PCR-free ultra-deep WGS, Chan et al. first identified the presence of foetus-associated cell-free DNA preferred ends at plasma from pregnant women [70]. They scanned the genome to check if certain locations had a significantly increased probability of being an ending position of plasma DNA fragments using a Poisson probability function. The ratio between foetal-specific preferred end and maternal-specific preferred end showed a high correlation with the foetal fraction estimated by the fraction of reads mapped to the chrY. Moreover, the fragments with these foetus-specific preferred ends showed similar fragment-size distributions as those overlapped with foetus-specific alleles. These observations disappeared at fragments that ended more than just 5 bp away from the foetal-specific preferred ends, which suggested the high specificity of these preferred-end positions from the foetus. Later, Jiang et al. discovered the liver-associated preferred ends at patients who received liver transplants and tumour-associated preferred DNA ends at cancer patients with a similar strategy [71]. In the follow-up study from the same group, Sun et al. suggested that the preferred-end positions were highly correlated with the nucleosomal structure [82]. They found that foetal preferred end sites were generally located in the nucleosome cores, while the maternal ones were located in the linker regions, which explained the relative shortness of foetal DNA in maternal plasma.

### The end-motif frequency and MDS [72]

Jiang et al. measured the end-motif frequency by calculating the frequency of the first 4-nucleotide (i.e. 4-mer) sequence on each 5′-fragment end of plasma DNA after alignment to the reference genome. The comparison of end-motif frequency revealed the significant differences between hepatocellular carcinoma (HCC) patients and healthy at some of the motifs across all the 256 combinations. For example, the CCCA motif showed significant reductions at the cfDNA from cancer patients compared with the non-malignant controls. Jiang et al. further utilised the normalised Shannon entropy to summarise the variations of the motif frequency into a single MDS score for each cfDNA WGS sample. A higher MDS value suggested a higher variety of plasma DNA molecules with different end motifs, while a lower MDS value indicated fewer varieties of plasma DNA end motifs. They found a higher MDS value in HCC patients than the non-malignant controls and utilised it as a marker for the diagnosis of HCC. At the same dataset, the MDS value showed a better classification power than other fragmentomic metrics, such as fragment size or OCF value developed in the same group. More importantly, by utilising the DNASE1L3-deleted model system in mice, they discovered that the DNASE1L3 might have a major role in generating the CC motif fragments predominant in cfDNA. In the human cancer data from the TCGA research network, they observed that the expression levels of DNASE1L3 across multiple cancers were generally downregulated. In several other additional literature, homozygous loss-of-function mutations of DNASE1L3 in both mouse model and human were found to significantly impact the end-motif frequency [83, 84]. These findings finally open the door to dissect the molecular mechanism behind cfDNA fragmentomics.

### Jagged end [73, 74]

The traditional WGS library-preparation step usually contains the DNA end-repair steps before the generation of NGS libraries, which erases the protruding-end (i.e. jagged-end) information. Early in 2008, Suzuki et al. identified the 5′ jagged ends in plasma DNA but not in gDNA by using the radioactive end-labelling procedure with the enzyme Klenow fragment [73]. CpG dinucleotides outside the CpG island in the human genome are usually methylated. In the library-preparation step of bisulfite sequencing, unmethylated cytosine is usually used for the end-repair step. Therefore, the uneven methylation status of CpG between the original and newly synthesised strand at the 3′ end may reflect the length of the jagged end. By utilising the publicly available cfDNA and gDNA WGBS data, Jiang et al. showed the proof-of-concept results of the genome-wide presence of jagged end at plasma DNA but not at sonicated genomic DNA from blood cells [74]. They developed a jag index (JI-U, Jagged Index-Unmethylated) in read 2 to measure the jaggedness of the fragments. The JI-U in cfDNA fragments between 130 and 160 bp showed a high performance in diagnosing the HCC in their previous cfDNA WGBS cohort. CpG dinucleotides are underrepresented in the human genome, which will affect the accuracy of measuring the length of the jagged end. Since non-CpG cytosine is usually unmethylated and widely distributed in the human genome, they further developed CC-tag technology by using methylated cytosine instead of unmethylated cytosine for the end-repair step. Therefore, in the context of CpH, they can utilise the JI-M (Jagged Index-Methylated) to characterise the jaggedness with higher accuracy. Finally, they observed the increases of jaggedness in mice with the deletion of DNASE1L3 (DNASE1L3−/−) compared with wild-type mice, whereas the decreases of jaggedness in mice with deletion of DNASE1 (DNASE1−/−), further revealed the possible biological mechanism behind jaggedness.

### CfDNA-fragmentation patterns at eccDNA [76–78]

Zhu et al. and Kumar et al. published the articles almost the same time about the genome-wide identifications of extrachromosomal circular DNA (eccDNA) at the cfDNA from human blood [76, 77]. To enrich eccDNA from cfDNA, Zhu et al. utilised the ATP-dependent DNase to selectively digest linear DNA and then applied the multiple-displacement-amplification (MDA) method to amplify the remaining circular DNA preferentially. Kumar et al. utilised the proteinase and exonucleases to remove linear DNA and subjected to rolling-circle amplification to increase the yield of circular DNA. Very recently, Sin et al. utilised exonuclease V to digest the background linear DNA and follow-up with MspI digestion or Tn5 tagmentation to build the NGS library [78]. These results suggested that ecc-cfDNA showed predominate peaks at dinucleosomal fragment size and highly enriched in exons, 3′UTR and CpG island, as well as the DNase-hypersensitive sites, H3K4Me1 and H3K27Ac marks. The ecc-cfDNA was also identified at cfDNA from cancer patients with longer fragment size than that from healthy controls, which suggested it as a potential biomarker for the disease diagnosis.

Overall, different approaches emerged rapidly to measure the cfDNA fragmentomics and even benefit other non-fragmentation-based measurements of cfDNA. For example, the characteristics of small fragment sizes from ctDNA or foetus have been utilised to enrich the ctDNA or cell-free foetal DNA (cffDNA) and thus increase the performance of cancer diagnosis and NIPT by CNVs [85–88]. Moreover, the shorter fragment size in ctDNA was utilised to filter out the possible clonal haematopoiesis of indeterminate-potential (CHIP)-associated genetic variants and improve the classification performance for the genetic-based approaches [89, 90]. The non-random fragmentation and fragment-size information have recently been incorporated into the mutational calling algorithm specifically designed for the cfDNA [91].

## Identification of tissues-of-origin by cfDNA fragmentomics

Different measurements of cfDNA fragmentomics described above are highly correlated with local and global epigenetic backgrounds, the patterns of which are known to be highly cell-type specific [92–94]. Therefore, it is possible to evaluate the tissues-of-origin by cfDNA fragmentomics.

DELFI, coverage near TFBS and cfDNA-fragmentation hotspots showed the potential to distinguish different cancer types in a supervised manner of machine learning but without providing the most relevant cell types contributed to cfDNA [64, 66, 68]. Preferred-end position, ended motif frequency and jagged end showed the potential for the estimation of the most relevant cell types but only demonstrated their correlations with the foetal sources in pregnant women, transplanted tissue source in organ- transplantation patients and tumour sources in cancer patients [70–72, 74].

Snyder et al. performed the fast Fourier transformation (FFT) on WPS signals in the first 10 kb of gene bodies [33]. Then they evaluated the correlations of the intensity of FFT signal against 76 gene expression datasets of human cell lines and primary tissues and found out that the most negatively correlated cell lines are haematopoietic lineages in healthy individuals. The intensity of FFT signals from the late-stage cancer patients showed the most positive correlations with cell lines from non-haematopoietic lineages. However, this WPS-based approach does not give the relative contributions from each cell type.

Sun et al. utilised OCF value around tissue-specific open-chromatin regions to solve this problem [67]. In healthy subjects, they observed the positive OCF values on open-chromatin regions from T cells and liver, and near or below zero on other tissue-specific open-chromatin regions. At cfDNA from pregnant women, liver transplantation and HCC patients, lung-cancer patients and colorectal-cancer patients, they observed the elevated OCF values on the placenta, liver, lungs or small intestine-specific open- chromatin regions, respectively. More importantly, the rank of the OCF value seems to provide the relative contributions from each cell type.

Liu et al. tried to estimate the absolute fractions of cell types that contributed to cfDNA by using A/B compartments inferred from FREE-C, which were represented as the linear combinations of 65 datasets from 18 tissues/cell types (Hi-C, H3K4me1 or WGBS data) [65]. However, the reference panel here is arbitrary and not completed yet, especially for the Hi-C data from different cell types. The inferred DNA methylation level from cfDNA seems to take advantage of the rich reference panel from DNA methylation and current well-established methylation-deconvolution methods to obtain the absolute contribution value from different cell types [69]. However, the inference accuracy of the methylation level in this approach is still not high enough for a robust and sensitive estimation of tissues-of-origin.

Overall, unlike the tissue-of-origin studies by cfDNA methylation in different pathological conditions [28, 95–105], the current tissue-of-origin analysis methods by cfDNA fragmentomics are still in their infant stage. An accurate and robust computational approach is still needed. Importantly, circulating cfDNA fragmentomics data from cancer patients contain the fragmentation information at DNA released from multiple cell types. A well-designed gold-standard system is needed for the complete benchmark and evaluation of the current tissue-of-origin analysis methods, including those based on cfDNA fragmentomics and methylation. Moreover, this is especially crucial in oncology for the fragmentation analysis in diagnosis and screening of cancers, given the presence of cfDNA deriving from a mixture of malignant, tumour-microenvironment and normal cells.

## Discussion

In summary, the cfDNA fragmentomics showed the potential to characterise different molecular layers, especially epigenetics, in gene regulation within the cells. Therefore, circulating cfDNA in the peripheral blood offers a promising non-invasive approach to monitor the dynamics of genetics and epigenetics information together from multiple cell types in a single WGS experiment. CfDNA in peripheral blood is mainly from haematopoietic cells in healthy people and additional diseased tissues in patients [28, 44, 95]. Thus, cfDNA fragmentomics may inform the initiation and progression of complex diseases such as early-stage cancers and autoimmune diseases that are associated with genetic and epigenetic aberrations in both primarily affected tissues as well as multiple immune cells [106–108]. Moreover, cfDNA-fragmentomics study in cancer does not suffer the problem of CHIP-associated genetic variations, which are not specific to cancer but a normal phenotype of ageing [109–111].

Beyond the circulating blood, cfDNA also existed in many other bodily fluids, such as urine and cerebrospinal fluid (CSF) [112–116]. The cfDNA fragmentomics showed significantly different patterns at these bodily fluids. The comprehensive characterisation of cfDNA fragmentation and the related biological mechanisms at these bodily fluids are still largely unexplored. More interestingly, how the cfDNA fragmentomics evolves in the context of evolution across different species beyond the mice is still not known.

Circulating cfDNA showed different fragmentation patterns when released from cellular apoptosis or necrosis. Neutrophil extracellular DNA traps (NETs) were identified in 2004 [117]. It has been shown that NETosis may lead to the release of DNA without the cell lysing upon certain conditions [56, 118–120]. How does NETosis affect the fragmentation patterns, especially with the presence of different pathogens, will be an interesting question to explore.

Currently, some urgent computational and experimental-related questions need to be solved for cfDNA fragmentomics. For example, the cfDNA yield and fragment sizes varied significantly with different pre-analytical steps, for instance, the cfDNA-extraction kits [121]. The fragmentation patterns in plasma cfDNA will also be severely affected by the contamination of genomic DNA from white blood cells, which could be due to the delay between blood draw and plasma preparation, storage time, plasma centrifugation and preparation approach, and different storage tubes [122, 123]. How to compare the cfDNA fragmentomics at samples generated from different protocols is a big challenge. Moreover, the fragment coverage and sizes measured by NGS are known to be affected by PCR amplification, G + C% content, mappability and k-mer composition [33, 124, 125]. Several computational approaches have been proposed and applied to correct or normalise these technical artefacts for the coverage and sizes [33, 64, 124–126]. However, extensive benchmark and comparison is still needed to find the optimal approach for different fragmentomic analysis at different resolutions. For instance, whether or not these technical factors will affect the other fragmentomic approaches, such as the preferred end and end motif, is still not well understood. Further, the batch effect is the major hurdle for applying cfDNA fragmentomics in disease diagnosis and prognosis [127]. Research groups began to evaluate the importance of PCR-amplification bias for the application of DELFI on their follow-up cancer screening study [126]. The performance of most approaches mentioned in this review was evaluated by cross-validation from the same dataset. Whether or not the trained machine-learning model in one dataset can still be applied to other independent datasets is a challenging problem, especially for the multiple different measurements across different genomic resolutions. Moreover, a lot of fragmentation-based approaches were developed recently and need more follow-up studies from different labs to replicate their power in the prospective clinical studies. Although with some recent progress, such as cfDNApipe [128], the lack of well-documented and well-maintained open-source bioinformatic packages for cfDNA fragmentomics is still a hurdle for the field [57]. Finally, a limited number of fragmentomic features were integrated together to represent the overall fragmentation level, such as WPS to integrate fragment coverage and end [33]. However, unlike the integration-method development in single-cell multi-omics field, it is urgently needed in the cfDNA field that how to integrate multiple cfDNA fragmentomic features in the same dataset, as well as other cfDNA signals [22, 25, 26, 129, 130], and therefore boost the power for the disease diagnosis. Several recent efforts on the multi-modality integration across different resolutions of fragmentomics and with other measurements began to show their power on the cancer diagnosis, such as LIQUORICE and others [85, 89, 90, 126, 131].

In 2020, the American College of Obstetricians and Gynecologists’ (ACOG) guidelines recommended the NIPT for all pregnancies, regardless of risk, which could eventually generate millions of cfDNA WGS every year in the United States. Moreover, large-scale cfDNA WGS datasets have already been generated for cancer detection and many other purposes [132]. However, most cfDNA WGS datasets are deposited in the controlled-access repositories for the purpose of protecting patients’ genotype information, which is not needed for the cfDNA fragmentomic analysis. Due to the enormous commercial interests behind the cfDNA, the access of raw cfDNA NGS data in these repositories usually requires data-transfer agreements that may take several months of negotiations between the two organisations’ legal departments. Some initial efforts, such as FinaleDB, have been made recently to establish the cfDNA-fragmentomics database [133–135]. However, more community efforts are still needed to collect and uniformly process the comprehensive publicly available cfDNA datasets together with their rich metadata. A centralised and uniformly processed cfDNA-fragmentomics database, similar to the ENCODE [136], will finally benefit this community in the long run.

In the end, a large number of cfDNA WGS and their associated clinical metadata from individuals at different physiological conditions will eventually allow us to characterise the baseline of the cfDNA fragmentomics and their variations in the population level at both healthy and pathological conditions.

## Data Availability

Not applicable.
